# Adolescent Mental Health in the Age of Social Media: Clinical Risks and Policy Lessons From Australia’s World-First Under-16 Restrictions

**DOI:** 10.7759/cureus.110808

**Published:** 2026-06-14

**Authors:** Enoch Chi Ngai Lim, Nga Chong Lisa Cheng, Chi Eung Danforn Lim

**Affiliations:** 1 Translational Research Department, Specialist Medical Services Group, Earlwood, AUS; 2 Data Science Institute, University of Technology, Sydney, AUS; 3 NICM (National Institute of Complementary Medicine) Health Research Institute, Western Sydney University, Westmead, AUS

**Keywords:** adolescent mental health, age assurance, australia, cyberbullying, digital health, social media, youth wellbeing

## Abstract

Social media is now embedded in adolescent relationships, identity formation, sleep, learning, emotion regulation, and help-seeking. This clinically oriented narrative review synthesizes evidence on adolescent social media use and mental health, with emphasis on anxiety and depression, sleep, cyberbullying, body image and eating concerns, self-harm-related content, digital social support, family context, and policy responses. The review aims to provide clinicians with a practical framework for assessment and management while using Australia’s under-16 social media restrictions as a policy case study. Current evidence does not support a simple screen-time model. Risk appears more closely related to adolescent vulnerability, harmful or algorithmically amplified content, compulsive or loss-of-control use, cyberbullying, sleep disruption, social comparison, limited coping strategies, and the availability of offline protective relationships. Australia’s Online Safety Amendment (Social Media Minimum Age) Act 2024, in effect from 10 December 2025, creates obligations for age-restricted platforms to take reasonable steps to prevent Australians under 16 from creating or maintaining accounts. This law should be understood as a platform-accountability intervention rather than a complete mental health intervention. Clinicians should assess social media use through functional impairment, sleep, mood, exposure to harmful content, cyberbullying, body image, self-harm risk, family media practices, and supportive online connections. Management should escalate when disclosures involve suicidal ideation, self-harm content, coercion, threats, image-based abuse, or severe sleep or eating disturbance. The goal is neither moral panic nor technological complacency, but safer digital environments and stronger family, clinical, school, and policy supports around young people.

## Introduction and background

Introduction

Digital media are now integral to the developmental environment of childhood and adolescence. Discussions of digital media exposure often focus on potential harm, but the mental health effects of technology are more complex than a simple risk model. Research on video games, play-based learning, and digital therapeutics suggests that interactive digital environments can have harmful or therapeutic effects depending on design, exposure, developmental stage, and therapeutic context [1]. This complexity is important when discussing social media. Social media is not simply “screen time”; it is a social, economic, commercial, algorithmic, and identity-forming space.

For this review, “adolescent” refers primarily to people aged 10 to 19 years, consistent with the World Health Organization definition used in adolescent health reporting [2]. “Social media” refers to internet-based platforms whose significant purpose includes user-generated content, social interaction, networking, recommendation, or communication. “Problematic” or “addictive” social media use refers not to high duration alone, but to patterns such as preoccupation, loss of control, withdrawal-like distress, deception or conflict about use, and interference with sleep, school, relationships, mood, or physical activity [3-5].

Social media and mental health are closely intertwined. The mental health of young people is a global concern. The WHO estimates that 1 in 7 people aged 10 to 19 years has a mental disorder [2]. Depression, anxiety, and behavior disorders are among the most common disorders in this age group [2]. Social media also intersects with emotion regulation, peer belonging, autonomy, social identity, body image, and sleep patterns.

According to the U.S. Surgeon General’s Advisory on Social Media and Youth Mental Health, social media use is common among young people, and there is not yet sufficient evidence to conclude that social media is safe for minors [6]. In a systematic review and meta-analysis, Fassi and colleagues examined the relationship between social media use and internalizing symptoms. They reported a positive association in clinical and community samples of adolescents, while also noting the limited number of studies in clinical cohorts [7]. The 2023 Youth Risk Behavior Survey from the Centers for Disease Control and Prevention reported that frequent social media use was observed in more than three-quarters of surveyed students and was associated with bullying, persistent sadness or hopelessness, and suicidal behavior [8]. More recent longitudinal research by Xiao and colleagues moved beyond total screen time by examining social media, mobile phone, and video game use across trajectories of increasingly addictive screen use. Increasing addictive screen-use trajectories were associated with mental distress, suicidal behavior, and suicidal ideation [3].

Recent recommendations also move away from simplistic time-based rules. The American Academy of Pediatrics situates children’s technology use within a “digital ecosystem” that includes algorithmic and artificial intelligence design, platform architecture, real-world and virtual communities, relationships, caregiver practices, environmental supports, and policy frameworks [4]. Findings from Pew Research Center show that adolescents report both positive social media experiences, such as connection and creative expression, and negative experiences, including effects on mental health, social confidence, sleep, and overwhelm [9]. The American Psychological Association similarly states that social media is not inherently positive or negative; its effects depend on platform features, content, the individual adolescent, and social context [5]. This variability suggests that clinical assessment should include online activities, their effects on daily life, family and home media practices, and the availability of offline support.

Australia has rapidly become a primary case study of social media policy. The Online Safety Amendment (Social Media Minimum Age) Act 2024 creates a legal obligation for Australian social media companies operating age-restricted platforms to take reasonable steps to ensure that Australians under 16 do not create or maintain a user account [10]. The eSafety Commissioner describes these as world-first restrictions and states that, as of 10 December 2025, platforms including Facebook, Instagram, Snapchat, TikTok, X, YouTube, Reddit, Twitch, Kick, and Threads must prevent Australians under 16 from maintaining user accounts [11]. The restrictions are often described publicly as a “ban,” but the legal mechanism is more specific: it imposes obligations on platforms, and not a criminal offence on children or parents [10,11].

This review addresses the existing evidence, the risks and benefits, the Australian legal and policy environment, and the implications for clinicians who work with adolescents and their families. Its main aim is to translate a broad and heterogeneous evidence base into a practical clinical and policy framework, rather than to estimate a single causal effect of social media exposure or to provide a quantitative synthesis.

Methods

This is a narrative review of peer-reviewed publications, public health guidance, professional society statements, and relevant policy documents concerning social media and adolescent mental health. Searches were conducted in PubMed and Google Scholar between 1 May and 31 May 2026 and updated on 12 June 2026. Professional society, government, and public health websites were checked during the same period, including WHO, CDC, the Office of the U.S. Surgeon General, the American Academy of Pediatrics, the American Psychological Association, Pew Research Center, the Australian Federal Register of Legislation, the eSafety Commissioner, and the Australian Government Department of Infrastructure, Transport, Regional Development, Communications, Sport and the Arts.

Representative PubMed search strategies included: (“social media” OR “social networking” OR “digital media”) AND (adolescen* OR teen* OR youth) AND (“mental health” OR depression OR anxiety OR suicid* OR “self-harm” OR sleep OR “body image” OR “eating disorder*” OR cyberbullying); (“problematic social media use” OR “social media addiction” OR “addictive screen use”) AND (adolescen* OR youth); (“social media” AND LGBTQ OR “sexual minority” OR “gender minority”) AND (adolescent OR youth); and (“age assurance” OR “age verification” OR “minimum age” OR “social media minimum age”) AND Australia. Google Scholar and website searches used equivalent combinations of these terms, including “Australia social media minimum age,” “under 16 social media restrictions,” “digital media policy,” and “age assurance technology trial.”

English-language sources published from 2020 to June 2026 were prioritized. Older or foundational sources were included when directly relevant to definitions, clinical assessment, or policy context. Eligible sources included systematic reviews and meta-analyses, longitudinal or trajectory studies, large national surveys, clinical and professional guidance, and official legislation or regulatory guidance. Sources were included when they addressed adolescents aged approximately 10 to 19 years, or youth samples that included adolescents, and when they provided evidence relevant to mental health, sleep, cyberbullying, body image or eating concerns, self-harm, supportive online communities, problematic or addictive use, family context, or social media policy.

Sources were excluded if they were opinion pieces, news reports, adult-only studies without adolescent relevance, studies focused solely on gaming or general digital therapeutics without relevance to social media, studies reporting only duration of use without mental health or safety outcomes, duplicate reports, or sources that did not clearly support a clinical or policy claim made in the review. Titles, abstracts, source summaries, and full texts where available were screened for relevance. When multiple sources addressed the same issue, priority was given to recent systematic reviews or meta-analyses, longitudinal evidence, large surveys, and authoritative guidance. The final evidence body was selected to support each clinical pathway and policy claim; direct evidence was added for sleep, body image and eating concerns, cyberbullying and self-harm, LGBTQ+ youth, neurodevelopmental vulnerability, parenting and family context, and age assurance.

No pooled effect sizes were calculated, no formal risk-of-bias assessment was performed, and no PRISMA flow diagram was produced because this was designed as a clinically focused narrative review rather than a systematic review or meta-analysis. To calibrate readers’ certainty, the tables identify the type of evidence supporting each clinical pathway and distinguish among systematic review evidence, longitudinal evidence, cross-sectional survey evidence, policy guidance, and expert or professional guidance.

## Review

Results

Overall, the literature suggests that social media is clinically relevant to adolescent mental health, but the relationship cannot be described by screen time alone. Across public health guidance, observational research, systematic reviews, and professional statements, risk appears to reflect the interaction of platform design and content, developmental stage and vulnerability, compulsive use, sleep displacement, peer relationships, family environment, and offline protective relationships [3-8,12,13]. Social media can contribute to distress through social comparison, cyberbullying, appearance-related pressure, exposure to negative content, and disrupted sleep; it can also support connection, creative expression, identity development, and help-seeking [4,5,9,14].

The evidence base varies by pathway. Associations between social media use and internalizing symptoms are supported by systematic review and meta-analytic evidence, although heterogeneity remains high and causality is difficult to infer [7,12]. Sleep disruption is supported by systematic review evidence linking social media use, including problematic use, to sleep problems [12]. Body image and eating concerns are supported by systematic review and meta-analysis of social comparison, body image concerns, and eating disorder symptoms [15]. Cyberbullying has longitudinal evidence linking exposure with suicidal ideation, attempted suicide, self-harm, and nonsuicidal self-injury, although findings differ by outcome and study design [16]. Evidence for supportive online connection is strongest for some groups, including LGBTQ+ youth, where social media can provide identity exploration, peer connection, and social support while also exposing young people to online harm [14].

Evidence also suggests that the association should be clinically differentiated. Adolescents who primarily use social media for communication, creativity, and positive peer engagement may not face the same risks as those whose use is compulsive, secretive, distressing, and functionally impairing. Increasing addictive screen-use trajectories are associated with greater risk of suicidal ideation, suicidal behavior, and mental distress [3]. This finding shifts attention from measured exposure to loss of control, distress, and interference with sleep, school, and relationships. Professional recommendations from the American Academy of Pediatrics (AAP) and American Psychological Association (APA) similarly emphasize family context, platform design, adolescent vulnerability, and digital media literacy rather than time limits alone [4,5].

Table 1 lists the clinical pathways linking social media use and adolescent mental health.

**Table 1:** Clinical pathways linking social media use and adolescent mental health

Pathway	Potential mechanism	Clinical signals to assess	Protective factors	Evidence type/supporting references
Internalizing symptoms	Social comparison, rejection sensitivity, exposure to distressing content, reinforcement of negative self-appraisal	Low mood, anxiety, rumination, withdrawal, reduced self-worth	Supportive peer relationships, emotion regulation skills, family communication, therapy access	Systematic review/meta-analysis, longitudinal study, survey and professional guidance [3,5-8,12]
Sleep disruption	Nighttime use, notifications, fear of missing out, emotionally arousing content, delayed bedtime	Short sleep, fatigue, school impairment, irritability, morning phone checking	Device-free bedtime routine, family media plan, parental modeling	Systematic review/meta-analysis and professional guidance [3-6,12]
Cyberbullying and social threat	Persistent peer conflict, public humiliation, exclusion, harassment, image-based abuse	School avoidance, panic symptoms, shame, secrecy, self-harm thoughts	Trusted adult disclosure, school response, platform/eSafety reporting, safety planning	Longitudinal systematic review, population survey and guidance [4-6,8,16]
Body image and eating concerns	Appearance-based comparison, edited images, influencer culture, algorithmic reinforcement of weight or fitness content	Body dissatisfaction, restrictive eating, compulsive exercise, appearance checking	Media literacy, reduced appearance-focused feeds, clinician screening, eating-disorder referral when indicated	Systematic review/meta-analysis and guidance [4-6,15]
Problematic or addictive use	Loss of control, distress when unable to access platforms, interference with school, sleep, relationships, or mood	Failed attempts to cut down, irritability, functional impairment, escalating use	Collaborative boundaries, alternative routines, treatment of comorbid anxiety, depression, or ADHD symptoms	Longitudinal trajectory study, systematic reviews and guidance [3-5,12,17]
Support and identity development	Peer belonging, creative expression, access to mental health information, connection for isolated youth	Online communities used for support, identity exploration, help-seeking	Safe communities, digital literacy, trusted adults, access to professional care	Survey evidence, systematic review in LGBTQ+ youth and guidance [4,5,9,14]
Family and home environment	Parental modeling, family conflict or support, inconsistent rules, technoference, lack of shared expectations	Conflict about devices, secrecy, inconsistent boundaries, parent-child disconnection	Warm parent-child relationships, consistent rules, shared routines, adult modeling, open communication	Systematic review of parenting and problematic social media use plus guidance [4,5,13]

A consistent observation is that the quality and context of social media use appear to matter more than duration alone. This does not mean duration is irrelevant, because prolonged use may displace sleep, physical activity, study, family time, and offline friendships. However, duration should be interpreted in relation to function, content, timing, emotional effect, and loss of control [3-5,12].

The home environment is an important protective or risk-modifying context. A recent systematic review of parenting and problematic social media use found more consistent evidence for positive parent-child relationships and positive family climate than for restrictive, internet-specific parenting alone [13]. This supports clinical questions about parental modeling, shared routines, device-free sleep practices, family conflict about technology, and whether rules are developed collaboratively rather than imposed only reactively.

Sleep disruption is a major modifiable pathway between social media use and adolescent well-being. Nighttime use, notifications, emotionally arousing posts, and fear of missing out can delay bedtime and impair sleep quality. Poor sleep can impair concentration and worsen mood symptoms. Cyberbullying and online peer conflict may also be persistent and difficult for adolescents to escape. Clinicians should ask direct, nonjudgmental questions about online bullying, threats, image-based abuse, coercion, and pressure to share private content [4-6,8,12,16].

The literature also supports asking about positive social media use. Some adolescents, particularly those who feel isolated offline, use online communities for belonging, identity development, psychoeducation, emotional support, and help-seeking [4,5,9,14]. Although risk should be assessed carefully, it is inaccurate to assume that all social media use is harmful. Practical clinical assessment domains and initial responses are listed in Table 2.

**Table 2:** Suggested clinical assessment domains and initial clinical responses

Domain	Suggested clinical question	Why it matters	Initial clinical response if positive	Supporting references
Function	“Is social media affecting sleep, school, friendships, physical activity, or family life?”	Functional impairment is more clinically meaningful than duration alone	Assess severity, screen mood/anxiety/school functioning, agree on a practical reduction or safety plan, and review progress	[3-5,12]
Mood relationship	“Do you usually feel better, worse, or unchanged after using it?”	Helps distinguish connection from distress reinforcement	Identify triggering platforms or content; consider feed curation, muting/unfollowing, therapy, or follow-up if distress persists	[5,7,9,12]
Compulsion	“Have you tried to cut down but found it difficult?”	Identifies problematic or addictive use patterns	Frame as loss of control and functional impairment; involve family in collaborative boundaries; treat comorbid anxiety, depression, or ADHD symptoms	[3-5,17]
Content exposure	“Are you seeing content about self-harm, extreme dieting, bullying, violence, or hate?”	Harmful content may worsen vulnerability or normalize risk behaviors	Assess self-harm or suicide risk, eating-disorder symptoms, and distress; report harmful content; escalate urgently if there is plan, intent, coercion, or immediate risk	[4-6,15,18]
Cyberbullying	“Has anyone threatened, humiliated, excluded, or pressured you online?”	Online peer harm may persist beyond school hours and affect safety	Assess immediate safety; document what happened; involve caregivers/school where appropriate; use platform and eSafety reporting; consider police if threats, extortion, or image-based abuse are present	[5,6,8,16,19]
Sleep	“Where is your phone at night, and do notifications wake you?”	Sleep disruption is a modifiable pathway to mood and learning problems	Use device-free bedroom routines, do-not-disturb settings, charging outside the bedroom, and treatment for insomnia, anxiety or depression where indicated	[4-6,12]
Body image/eating	“Does your feed affect how you feel about your body, food, exercise, or weight?”	Appearance-focused content may reinforce dissatisfaction and disordered eating	Screen for weight loss, restriction, bingeing, purging, compulsive exercise and medical instability; refer to eating-disorder services when indicated	[4-6,15]
Protective use	“Are there online spaces that make you feel supported or less alone?”	Avoids assuming all use is harmful and identifies safe supports	Preserve safe and affirming supports while reducing harmful exposure; connect online support to trusted offline adults and professional care	[4,5,9,14]
Family context	“What rules do adults use, and do they follow them too?”	Parental modeling, consistency and family climate influence adolescent behavior	Discuss adult modeling, family conflict, co-viewing, shared expectations and routines; avoid purely punitive or reactive restrictions	[4,5,13]

When assessment identifies self-harm content, suicidal thoughts, threats, coercion, image-based abuse, or severe sleep or eating disturbance, clinicians should move beyond advice about screen time. The response should include assessment of immediate safety, suicidal ideation, plan, intent and access to means; a developmentally appropriate safety plan; involvement of caregivers unless doing so increases risk; consideration of safeguarding and confidentiality obligations; and urgent referral to emergency, crisis, child and adolescent mental health, or specialist eating-disorder services when clinically indicated [18]. For cyberbullying, sextortion, or image-based abuse, clinicians should advise adolescents not to retaliate or redistribute images, support evidence preservation where safe and lawful, use platform and eSafety reporting mechanisms, and involve schools or police where threats, exploitation, or immediate danger are present [19].

Policy literature and Australian regulations provide a broader perspective on this evidence. Australia’s under-16 social media restrictions are a large-scale platform-accountability intervention under the Online Safety Amendment (Social Media Minimum Age) Act 2024 [10]. The restrictions came into effect in Australia on 10 December 2025. The eSafety Commissioner describes them as world-first restrictions requiring age-restricted social media platforms to take reasonable steps to prevent Australians under 16 from creating or maintaining accounts [11]. The policy reflects concerns about children’s vulnerability to harmful content, cyberbullying, persuasive design, and compulsive use, as well as the difficulty parents face in setting boundaries when social media is widely normalized [4,5,10,11,13].

The policy should not be framed as a stand-alone mental health intervention. It does not replace family support, school mental health programs, clinical assessment, suicide prevention, eating-disorder care, cyberbullying response, or safer platform design. It also does not prevent all online exposure: eSafety notes that some services, including services whose sole or primary purpose is messaging or online gaming, age-appropriate education, health and support services, may remain available to under-16s [11].

Potential advantages of the Australian model include delaying account-based exposure during a sensitive developmental period, reducing social media-related distraction, supporting safer platform design, decreasing cyberbullying risk, and easing pressure on parents by shifting some compliance responsibility from individual families to platforms [4,5,10,11,13]. Potential limitations include displacement to less regulated services, uncertainty about platform compliance, privacy and equity concerns in age assurance, ineffective age restrictions, false classification, over-blocking, and the risk that policy will be mistaken for a complete mental health solution [4,5,10,11,20]. A summary of Australia’s under-16 restrictions is provided in Table 3.

**Table 3:** Australia’s under-16 social media restrictions: rationale, benefits, risks, and evaluation

Policy dimension	What the policy does	Potential benefit	Potential risk or limitation	Evaluation priorities
Minimum access age	Requires age-restricted platforms to take reasonable steps to prevent Australians under 16 from creating or keeping accounts	Delays account-based exposure during a sensitive developmental period	Age alone may not capture maturity, vulnerability, or family context	Monitor mental health, sleep, cyberbullying, help-seeking, and youth-reported wellbeing before and after implementation
Platform accountability	Shifts part of compliance responsibility from families to industry systems	Reduces reliance on individual parents to enforce unpopular boundaries	Enforcement may be uneven across platforms and services	Require transparent compliance reporting, independent audit, and assessment of account removal and appeals
Harmful design features	Creates pressure to address engagement-based features such as notifications, recommender systems, autoplay, and infinite scroll	Encourages safer design and public scrutiny	Young people may migrate to less regulated, encrypted, gaming, or private spaces	Track displacement, VPN use, account sharing, and movement to excluded or emerging services
Age assurance	Platforms need practical methods to identify underage users and manage disputes	May improve compliance beyond self-declared age	Privacy concerns, false positives, false negatives, demographic bias, over-blocking and exclusion may arise	Evaluate accuracy near the age threshold, demographic fairness, data minimization, appeal outcomes, and privacy incidents
Supportive resources	The law targets accounts on age-restricted platforms, not all online support or information	May reduce harmful exposure while allowing access to health, education and support services	Could restrict access to affirming communities and mental health information if implemented too broadly	Assess access for rural, isolated, LGBTQ+, neurodivergent and marginalized adolescents
Public health signalling	Creates a national norm that social media access is not developmentally neutral	Supports families and schools in setting boundaries	May reduce investment in clinical, school-based, family, or community support if treated as a complete solution	Evaluate family functioning, school reports, clinical presentations, and whether complementary services are strengthened

More concrete policy evaluation is needed. Mental health outcomes should include depressive and anxiety symptoms, sleep quality, self-harm presentations, suicidal ideation, cyberbullying, eating-disorder risk, social connectedness, help-seeking, and school functioning. Equity and privacy outcomes should include age-assurance error rates near the threshold age, false classification, appeal timeliness, demographic bias, privacy-preserving design, data minimization, and user trust. Behavioral outcomes should include displacement to alternative services, non-account access, account sharing, VPN use, and migration to less regulated spaces. Family and school outcomes should include conflict about devices, parental workload, school reports of online harm, and whether families experience the law as supportive or punitive [11,20].

Discussion

From Screen Time to Function, Content, and Context

The main clinical shift is from counting hours to understanding function, content, timing, control, and context. Treating risk as a simple function of time online oversimplifies the issue [3-7,12]. Displacement of sleep, learning, physical activity, family time, or face-to-face relationships is a stronger clinical signal than duration alone [3-6,12]. Assessment should therefore determine whether use is functional, compulsive, supportive, or harmful [3-5,9,14]. Two adolescents may spend the same amount of time online but have very different experiences: one may be creating content and maintaining supportive relationships, whereas another may be caught in cycles of scrolling, comparison, rejection, or bullying [4,5,7,9].

A practical four-question framework remains useful. First, what does social media replace? Sleep, exercise, study, family time, and face-to-face friendships may be displaced [3-6,12]. Second, what does it amplify? Social media may intensify social comparison, anxiety, loneliness, body image concerns, or exposure to harmful content [4-8,15,16]. Third, what does it provide? Supportive networks may offer connection, creativity, identity exploration, psychoeducation, and help-seeking [4,5,9,14]. Finally, can the adolescent control it? Loss of control, distress when disconnected, failed attempts to reduce use, or functional impairment suggests problematic or addictive use [3-5,17].

Home Environment, Schools, and Health Systems

The role of the home environment is central. Families are not simply enforcement agents for platform rules; they provide the relational climate in which adolescents learn routines, boundaries, conflict resolution, emotional regulation, and help-seeking. Positive parent-child relationships, warm communication, consistent expectations, and adult modeling appear more protective than reactive restriction alone [4,13]. Clinicians should therefore ask how the family uses devices, whether rules are shared and consistent, whether there is conflict or secrecy, and whether adults model the same sleep and attention practices they ask of young people.

Schools are often where online harm first becomes visible. Social media-related conflict can contribute to distraction, bullying, exclusion, and safety concerns during and beyond school hours [4-6,8,16]. Digital literacy and online harm prevention should therefore be incorporated into school mental health promotion. Health systems should routinely address digital life as part of adolescent psychosocial assessment, including sleep, mood, cyberbullying, harmful content, body image, self-harm exposure, supportive online communities, and family rules [4-6,8,18].

Risks for Vulnerable Groups

Risk is not evenly distributed. Adolescents experiencing bullying, social exclusion, mental health symptoms, neurodevelopmental difficulties, body image concerns, family conflict, or limited offline support may be more vulnerable to harm from social media [3-8,13,15-17]. At the same time, online communities may provide affirmation and belonging for some isolated or marginalized adolescents, including LGBTQ+ youth [4,5,9,14]. Clinical screening should therefore be specific and proportional. A young person who is functioning well at school, maintaining offline relationships, and using social media for connection may not need the same intervention as one whose use is compulsive, secretive, distressing, sleep-disrupting, or linked to self-harm content, suicidal thoughts, coercion, or eating-disorder behaviors [3-6,8,15,16,18]. The goal is calibrated care rather than universal restriction.

Policy Implications

Australia’s under-16 social media restrictions represent a major shift in digital regulation by placing greater responsibility on platforms for young users’ safety [10,11]. The law may support families by reducing the pressure on individual parents to set boundaries in a context where social media access is normalized among peer groups [4,10,11,13]. However, the policy should also be considered in terms of unintended effects. Restrictions may lead some adolescents to migrate to less regulated services or private channels, and age-assurance processes may raise concerns about privacy, false classification, demographic bias, over-blocking, and equity [11,20]. Clinicians should therefore not treat age restriction as a substitute for mental health care, family support, school-based prevention, digital literacy, or safer platform design.

Limitations and future research

As this is a narrative review rather than a systematic review, it does not provide pooled effect-size estimates, formal risk-of-bias evaluations, or a PRISMA flow diagram. Although the methods have been expanded to include search dates, representative search strategies, eligibility criteria, and source selection rationale, source choice remains vulnerable to selection bias. The review also relies on a relatively small final set of references for a broad topic, even after adding targeted evidence for sleep, body image and eating concerns, cyberbullying and self-harm, LGBTQ+ youth, family context, ADHD-related vulnerability, clinical safeguarding, and age assurance.

The evidence is heterogeneous because studies differ in definitions of social media use, participant age, platforms, outcome variables, and follow-up duration [3,7,12]. Most included studies are observational and therefore provide limited evidence of causality [3,7,8,12]. Cross-sectional associations may reflect bidirectional or reverse relationships, including the possibility that adolescents experiencing distress use social media differently. Much of the evidence comes from high-income countries, which limits generalizability to lower-resource settings and culturally diverse populations. Social media platforms also evolve quickly, and older studies may not fully reflect current short-form video ecosystems, algorithmic recommendation systems, AI-mediated content, private messaging, or emerging synthetic media risks [4,20].

There is also limited direct post-implementation evidence from Australia’s under-16 restrictions. Future policy research should evaluate mental health outcomes, displacement to other services, privacy, equity, family functioning, school impacts, and community well-being [11,20]. Future clinical research should prioritize longitudinal and experimental designs that distinguish among platform type, content, use pattern, sleep displacement, compulsive use, peer context, family environment, and individual vulnerability [3,4,7,12,13,17]. Clinical studies are needed for adolescents with depression, anxiety, eating disorders, neurodevelopmental disorders, trauma, and marginalized identities. Young people, especially those from marginalized groups, should be involved in study and policy design [4,5,9,14].

## Conclusions

Social media is embedded in adolescent development and can influence mental health through platform design, content, family context, sleep, peer relationships, and individual vulnerability. The clinically useful question is not whether social media is universally harmful or beneficial, but how a particular adolescent uses it, what it replaces, what it amplifies, what it provides, and whether it can be controlled. Australia’s under-16 restrictions, now in effect, provide an important test of whether platform accountability can reduce harmful exposure while preserving access to safe support and information. The policy’s effects on mental health, equity, privacy, family functioning, and behavior should be monitored carefully. Clinicians should assess digital life routinely, respond promptly to high-risk disclosures, involve families and schools where appropriate, and help adolescents build safer digital habits alongside strong offline protective relationships.
